# The Predictive Value of Graft Viability and Bioenergetics Testing Towards the Outcome in Liver Transplantation

**DOI:** 10.3389/ti.2024.12380

**Published:** 2024-02-23

**Authors:** Andras T. Meszaros, Annemarie Weissenbacher, Melanie Schartner, Tim Egelseer-Bruendl, Martin Hermann, Jasmin Unterweger, Christa Mittelberger, Beatrix A. Reyer, Julia Hofmann, Bettina G. Zelger, Theresa Hautz, Thomas Resch, Christian Margreiter, Manuel Maglione, Timea Komlódi, Hanno Ulmer, Benno Cardini, Jakob Troppmair, Dietmar Öfner, Erich Gnaiger, Stefan Schneeberger, Rupert Oberhuber

**Affiliations:** ^1^ Department of Visceral, Transplant and Thoracic Surgery, Medical University of Innsbruck, Innsbruck, Austria; ^2^ Institute of Pathology, Neuropathology and Molecular Pathology, Medical University of Innsbruck, Innsbruck, Austria; ^3^ Oroboros Instruments, Innsbruck, Austria; ^4^ Department of Medical Statistics, Informatics and Health Economics, Medical University of Innsbruck, Innsbruck, Austria

**Keywords:** liver, transplantation, static cold storage, mitochondria, high-resolution respirometry, real-time confocal imaging

## Abstract

Donor organ biomarkers with sufficient predictive value in liver transplantation (LT) are lacking. We herein evaluate liver viability and mitochondrial bioenergetics for their predictive capacity towards the outcome in LT. We enrolled 43 consecutive patients undergoing LT. Liver biopsy samples taken upon arrival after static cold storage were assessed by histology, real-time confocal imaging analysis (RTCA), and high-resolution respirometry (HRR) for mitochondrial respiration of tissue homogenates. Early allograft dysfunction (EAD) served as primary endpoint. HRR data were analysed with a focus on the efficacy of ATP production or *P*-*L* control efficiency, calculated as 1-*L*/*P* from the capacity of oxidative phosphorylation *P* and non-phosphorylating respiration *L*. Twenty-two recipients experienced EAD. Pre-transplant histology was not predictive of EAD. The mean RTCA score was significantly lower in the EAD cohort (−0.75 ± 2.27) compared to the IF cohort (0.70 ± 2.08; *p* = 0.01), indicating decreased cell viability. *P*-*L* control efficiency was predictive of EAD (0.76 ± 0.06 in IF vs. 0.70 ± 0.08 in EAD-livers; *p* = 0.02) and correlated with the RTCA score. Both RTCA and *P*-*L* control efficiency in biopsy samples taken during cold storage have predictive capacity towards the outcome in LT. Therefore, RTCA and HRR should be considered for risk stratification, viability assessment, and bioenergetic testing in liver transplantation.

## Graphical Abstract

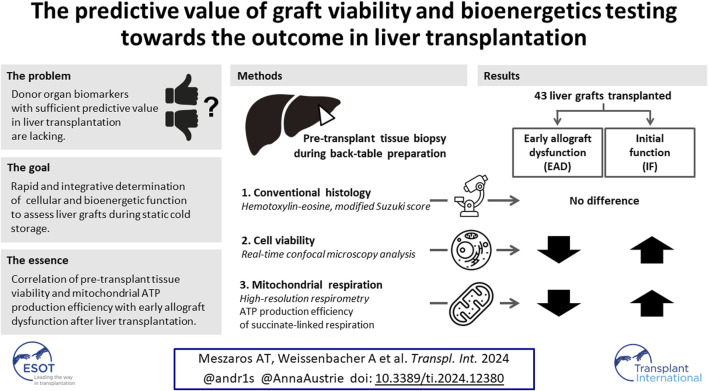

## Introduction

The limited number of organ donors and the low number of livers of deceased donors with optimal organ quality are key restricting factors in liver transplantation (LT). While the indications for LT are increasing, many technical aspects and tools for the assessment of graft quality have not changed [1, 2]. The outcomes have been improving steadily with LT survival rates reaching 90% after the first year [3], but up to 20% of patients are dying while waiting or being removed from the liver transplant waiting list due to the scarcity of available organs [2, 4–6]. Current efforts to enlarge the donor pool and to increase organ utilization is the inclusion of livers from extended criteria donors (ECD), from donors after circulatory death (DCD), steatotic organs, and livers with longer cold and warm ischemia times [1, 7, 8]. Accepting such pre-injured organs for LT is afflicted with an increased risk of morbidity and mortality [9–12]. The above grafts are more susceptible to temperature fluctuations, ischemia and re-oxygenation (ischemia-reperfusion injury, IRI). Furthermore, the activation of damage associated molecular pattern proteins (DAMPs) in the donor (as a response of brain death) during reperfusion results in secretion of proinflammatory cytokines triggering inflammation and consecutive damage of the liver [9, 13].

In addition to the inflammatory response, the impairment of mitochondrial function during IRI is considerable. Oxygen deprivation, ATP depletion, and the enhanced generation of reactive oxygen species (ROS) during reperfusion can alter the bioenergetic status and mitochondrial integrity [9, 13–15]. While many mechanisms contributing to IRI and the subsequent organ dysfunction are known, further details and their immediate clinical implications remain to be elucidated. Both the assessment of cell viability and bioenergetic function have merit in the search for biomarkers with such predictive value.

Previously, Martins *et al.* described a clear relationship between IRI and impaired mitochondrial respiration in liver transplantation. In a murine model, mild hypothermia was protective against loss of mitochondrial membrane potential [14]. A correlation was demonstrated in a clinical trial between LT and mitochondrial function, aminotransferase peaks, and arterial lactate levels [15]. More recently, a correlation between mitochondrial injury and the outcome in LT has been suggested. Hypothermic oxygenated machine perfusion (HOPE) may improve cellular bioenergetics and flavin mononucleotide (FMN) was proposed as a biomarker, a shedding product of the mitochondrial Complex I monitored in the perfusate during machine perfusion [16, 17]. While the analysis of metabolic products such as FMN may indicate mitochondrial damage, it does not allow for evaluation of the actual bioenergetic capacity.

In addition to the bioenergetic function, the assessment of cell viability and damage during and after LT may help to predict the fate of an organ. Real-time confocal analysis (RTCA) of tissue samples was found to have predictive value toward the occurrence of delayed graft function in kidney biopsies [18]. This method was validated in a murine liver warm ischemia model [19] and its applicability for characterization of cell viability during clinical liver NMP was recently demonstrated [20].

We have previously assessed mitochondrial respiration during normothermic machine perfusion (NMP) of the liver and found a predictive capacity towards the outcome after LT [20]. The aim of the present study was to evaluate the relevance and capacity of RTCA and HRR in clinical LT after static cold storage (SCS). We hypothesized that both cell viability assessment and evaluation of mitochondrial respiratory function provide integrative assessments of subcellular and cellular function and damage to liver grafts. Our goal was to employ methods for rapid assessment without the need for isolation of mitochondria or tissue fixation. Our results confirm a correlation between RTCA and mitochondrial function and the outcome after LT.

## Materials and Methods

### Clinical Trial Design

Based on a previously established technology with RTCA [19, 21, 22], a prospective, single arm, observational clinical trial was conducted at the Medical University of Innsbruck between October 2017 and October 2019. The study was approved by the institutional review board of the Medical University of Innsbruck (vote number 1025/2017). All patients participating in the trial signed the respective informed consent form.

All liver grafts stemmed from donors after brain death and none of the livers underwent machine perfusion.

Forty-three consecutive patients were included in this trial. Recipient, donor, and transplant characteristics were collected and collated. Early allograft dysfunction (EAD) served as primary endpoint, Model for Early Allograft Function (MEAF [23]) Liver Graft Assessment Following Transplantation (L-GrAFT [24, 25]), graft and patient survival, length of stay and biliary complications served as secondary endpoints. EAD was defined as the presence of one or more of i) bilirubin ≥10 mg·dL^−1^ on day seven after transplantation, ii) international normalized ratio (INR) ≥ 1.6 on day seven, and iii) alanine (ALT) or aspartate aminotransferases (AST) > 2000 IU·L^−1^ within the first 7 days after liver transplantation [26].

### Sampling and Preparing Liver Biopsies for Real-Time Live Confocal Imaging

Liver wedge biopsies were taken during the back-table preparation. All biopsy samples were placed in HTK solution (Custodiol^®^, Dr. Franz Köhler Chemie GmbH, Bensheim, Germany) on ice for transportation prior to analysis.

Real-time live confocal microscopy assessment was performed using the following live stains: Wheat germ agglutinin conjugate (WGA; Molecular Probes, Eugene, OR, United States; 10 μg·mL^−1^ final concentration) visualizes the tissue morphology, SYTO^®^16 (Molecular Probes; final concentration 5 µM) visualizes all nuclei and propidium iodide (PI) (Molecular Probes; final concentration 500 nM) the nuclei of dead cells [20]. Incubation time was 15 min at 37°C. Real-time live confocal imaging was performed in eight-well chambered cover glasses (Nalge Nunc International). Images were acquired with a spinning disk confocal system (UltraVIEW VoX; Perkin Elmer, Waltham, MA) connected to a Zeiss Axio Observer Z1 microscope (Zeiss, Oberkochen, Germany) and visualized employing the Volocity software (Perkin Elmer) using a ×10 objective. Time for readout was approximately 5 min per sample.

### High-Resolution Respirometry

High-resolution respirometry (HRR, O2k, Oroboros Instruments, Innsbruck, Austria) was applied to assess mitochondrial respiration. All measurements were carried out in O2k-chambers of 2 mL at 37°C under constant stirring at 750 rpm [27]. Data were acquired at intervals of 2 s and analysed with the DatLab software (Datlab 7.4, Oroboros Instruments, Innsbruck, Austria). Besides monthly instrumental background calibrations, before each experiment, air-calibration was performed with MiR05 mitochondrial respiration medium (MiR05-Kit, Oroboros Instruments, Innsbruck, Austria). The finally prepared medium consists of 0.5 mM EGTA, 3 mM MgCl_2_ • 6 H_2_O, 60 mM lactobionic acid, 20 mM taurine, 10 mM KH_2_PO_4_, 20 mM HEPES, 110 mM D-sucrose, 1 g·L^−1^ essentially fatty acid free bovine serum albumin. Twenty mg of liver tissue was dissected on a cooled plate at 4°C, weighted, and subsequently homogenized in 4°C MiR05 using a PBI-Shredder O2k-Set (Oroboros Instruments, Innsbruck, Austria) according to the manufacturer’s instructions. 2-mL tissue homogenate with a final concentration of 1 mg wet mass·mL^−1^ was immediately added into each of the O2k-chambers. Chemicals for the pre-defined substrate-uncoupler-inhibitor titration (SUIT) protocols were titrated using glass microsyringes (Oroboros Instruments, Innsbruck, Austria). The SUIT protocols (i; corresponding SUIT-025^1^) and (ii; corresponding SUIT-006 O2 mt D047^2^) are defined in the Supplementary Tables S1, S2. Each titration step was carried out after respiration reached a steady state. Measurements were performed in technical duplicates.

Respiration rates were expressed as O_2_ flux per wet mass tissue [pmol O_2_·s^−1^·mg^−1^].

Three substrate pathways delivering convergent electron flow to the electron transport system were investigated. The fatty acid oxidation (FAO)-pathway F was determined in the presence of octanoylcarnitine and a low concentration of malate, the NADH-pathway N with the substrates pyruvate, glutamate, and malate. The succinate-linked pathway S was assessed after inhibiting the mitochondrial Complex I with rotenone and adding succinate. In addition to studying these pathways separately, the combined pathways FNS feeding electrons into the coenzyme Q-junction were investigated to reconstitute the tricarboxylic acid cycle function of the living cell and determine possible additive effects [28]. For further details, see Supplementary Tables S1, S2.

Respiratory capacities were normalized to an internal reference rate for each measurement to determine flux control ratios (*FCR*) for evaluation of SUIT protocol (i). For SUIT protocol (ii) the coupling states LEAK (*L*), OXPHOS (*P*), and OXPHOS(c) (*Pc*), were evaluated [28]. LEAK, a dissipative component of respiration, was measured in the presence of the mitochondrial Complex I inhibitor rotenone and reducing substrate succinate without ADP (rate S_*L*_). The respiratory capacity of oxidative phosphorylation (OXPHOS) was assessed in the presence of succinate, 5 mM ADP, and 10 mM inorganic phosphate in MiR05 (S_*P*_). Finally, cytochrome *c* was added to test the integrity of the mitochondrial outer membrane, obtaining the rate S_*Pc*_. Based on the above, the following control efficiencies were calculated for the succinate pathway: *P*-*L* control efficiency (1-*L*/*P*), to evaluate the efficiency of ATP production in the succinate pathway, and cytochrome *c* control efficiency (*j*_*c*_ = 1-*P*/*Pc*) to evaluate the damage to the mitochondrial outer membrane [28].

### Real-Time Confocal Analysis

In each liver biopsy, 10 optical sections of 1 µm were analysed. Cell viability and matrix architecture of the liver were quantified by counting events (one event is either a viable or a non-viable cell) and groups which comprise i) total count of cells, irrespective of the localization; ii) cells from the central vein area; iii) cells from the portal triad area.

For each group, the number (total count) of viable cells was divided by the number of non-viable cells (total count) with following possible results: (+1) for highly viable biopsies/areas with more viable than non-viable cells; (0) for biopsies/areas in which the number of viable cells equals the one of non-viable cells; (−1) for those in which the number of non-viable cells outnumbers the one of viable cells. For each biopsy, a score was calculated consisting of two central vein areas and one portal triad area resulting in a maximum of +3 points in the best or −3 in the worst-case scenario.

### Histopathological Assessment

After completion of live confocal imaging, the liver biopsy was placed and fixed in Millonig’s solution and processed for paraffin embedding. Four µm thick sections were stained by haematoxylin and eosin as per standard protocols. Light microscopy observations were carried out on a Nikon Eclipse 50i microscope (Nikon Corporation, Japan). Histological assessment was performed according to a modified Suzuki score [29] based on necrosis, steatosis, inflammation, fibrosis and vascular changes (Supplementary Table S3). An overall histopathologic score indicated i) normal liver tissue or only mild histopathologic alterations—score 3 (subscores 0 or 1), ii) moderate histopathologic alterations—score 2 (at least one subscore 2), or iii) severe histopathologic alterations—score 1 (at least one subscore 3). Slide scanning was performed on an Olympus VS120 microscope and evaluated using Olympus OlyVIA software.

### Statistical Analysis

The statistical testing was done with Graph Pad Prism 9 and IBM^®^ SPSS^®^ Statistics Version 25. A *p*-value of <0.05 was considered as statistically significant. Biopsy results (RTCA, histology scores and HRR), recipient, donor and transplant factors were analysed using parametric and non-parametric tests (including Spearman rank correlation). The RTCA score and *P*-*L* control efficiency were adjusted for clinically relevant parameters and evaluated in uni- and multivariate logistic regression analyses.

## Results

### Patient Demographics and Early Allograft Dysfunction

Table 2 depicts the demographics and the transplant data of 43 liver transplants stratified for EAD (*N* = 22, 51.2%) and initial function (IF, *N* = 21) following liver transplantation. The proportion of ECD was numerically, but not statistically higher in the cohort developing EAD (18/22, 81.1%) compared to patients with IF (13/21, 61.9%), *p* = 0.27. Recipients with EAD received livers from donors with a significantly higher BMI (28.05 ± 6.29 kg·m^−2^ in EAD vs. 24.6 ± 4.16 kg·m^−2^ in IF, mean ± SD; *p* = 0.031). The Liver and the Eurotransplant donor risk indices (DRI) were comparable between the groups. The anastomosis time was significantly longer in EAD-patients compared to patients with IF livers (47.64 ± 9.86 min in EAD vs. 40.57 ± 5.92 min in IF-patients, *p* = 0.02). Patients developing EAD had significantly higher mean MEAF-scores (6.68 ± 1.3, compared to liver recipients with IF, 4.77 ± 1.41, *p* < 0.0001). The mean L-GrAFT score was −0.63 ± 1.13 and corresponded with EAD (−0.26 ± 1.21 in EAD vs. −1.11 ± 0.82 in IF, *p* = 0.015).

### Technical Feasibility

RTCA and scoring in fresh liver wedge biopsy samples collected from donor livers after static cold storage was completed in approximately 30 min. HRR took 90 min including sample preparation. Hence, the two methods which were carried out simultaneously proved to be feasible for immediate assessment albeit requiring availability of staff and respective expertise at the point in time. Both assessments required <40 mg tissue sample (wet mass), and, if the technology is available, have a cost per analysed sample of ca. 200 EUR.

### Characterization of Mitochondrial Function in Human Liver Samples

**FIGURE 1:**
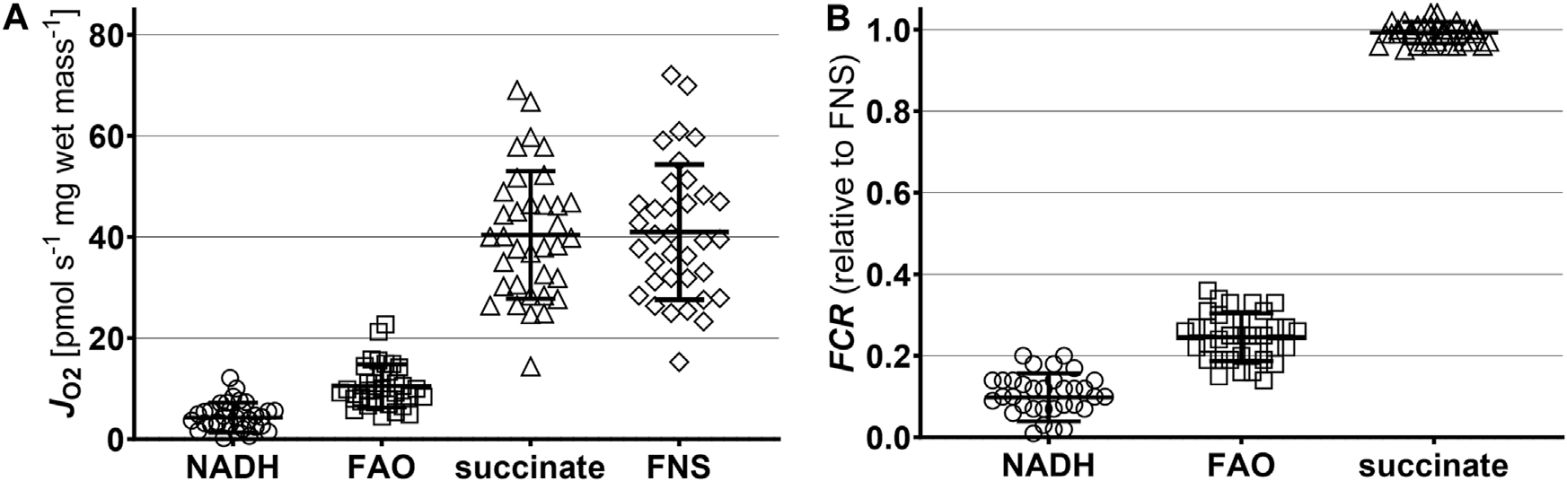
Pathway control analysis of mitochondrial respiration after static cold storage. **(A)** Respiration in the OXPHOS state with saturating ADP concentration (5 mM). Pyruvate (5 mM), malate (2 mM), and glutamate (10 mM) were the substrates for the N-linked pathway (NADH). Octanoylcarnitine (0.5 mM) and malate (0.1 mM) supported fatty acid oxidation (FAO). Rotenone (0.5 µM) and succinate (10 mM) were added to assess the S-linked pathway (succinate). FNS represents the OXPHOS capacity with all pathways converging at the Q-junction [28]. **(B)** Relative contributions of the three mitochondrial pathways, expressed as flux control ratios (*FCR*). *FCR* of the F-, N-, and S-linked pathways were calculated relative to the maximum OXPHOS capacity reached after addition of substrates of all three pathways. Results are shown as individual values, mean ± SD.

In a first step, we analysed the capacity of mitochondrial oxidative phosphorylation (OXPHOS) in liver crude homogenates for the NADH-linked, fatty acid oxidation (FAO), and succinate pathways (Figure 1A; Table 1). In the biopsies after SCS in the whole cohort (*N* = 43), respiration was highest for succinate-linked OXPHOS (40.4 ± 12.6 pmol·s^−1^·mg wet mass^−1^), with a considerable variation between grafts. In contrast, respiration was markedly lower for the FAO and NADH pathways (10.5 ± 4.3 and 4.3 ± 3.0 pmol·s^−1^·mg wet mass^−1^, respectively). No difference was found between the EAD and IF groups.

**TABLE 1:** High-resolution respirometry.

	EAD *(N* = 22)	IF (*N* = 21)	*p*-value
**Characteristics (mean ± SD)**			
**Pathway control: OXPHOS capacity per mg wet mass**			
FAO pathway F_*P*_ [pmol·s^−1^·mg^−1^]	10.75 ± 4.37	10.26 ± 4.23	ns
NADH pathway N_*P*_ [pmol·s^−1^·mg^−1^]	4.12 ± 2.88	4.43 ± 3.09	ns
Succinate pathway S_*P*_ [pmol·s^−1^·mg^−1^]	42.94 ± 12.62	40.04 ± 13.19	ns
Convergent pathway FNS_*P*_ [pmol·s^−1^·mg^−1^]	43.50 ± 13.25	40.61 ± 14.06	ns
FAO pathway *FCR* (relative to FNS)	0.25 ± 0.06	0.24 ± 0.06	ns
NADH pathway *FCR* (relative to FNS)	0.09 ± 0.06	0.11 ± 0.06	ns
Succinate pathway *FCR* (relative to FNS)	0.99 ± 0.03	0.99 ± 0.03	ns
**Coupling control: Succinate pathway characteristics**			
LEAK respiration S_*L*_ [pmol·s^−1^·mg^−1^]	12.07 ± 5.64	9.67 ± 3.67	ns
OXPHOS capacity S_*P*_ [pmol·s^−1^·mg^−1^]	40.76 ± 16.26	38.83 ± 11.19	0.94
Cytochrome *c* control efficiency, 1-S_*P*_/S_*Pc*_	0.17 ± 0.12	0.11 ± 0.09	0.12
***P-L* control efficiency, 1-S**_***L***_ **/S**_***P***_	**0.70 ± 0.08**	**0.76 ± 0.06**	**0.02**

Statistically significant differences are bold.

Next, we calculated the flux control ratios (*FCR*) as the single pathway capacities relative to the maximum OXPHOS respiration reached with the combination of all substrates. As shown in Figure 1B; Table 1, succinate-linked respiration alone was sufficient to saturate OXPHOS capacity. This pattern of pathway control reflects an incomplete additivity [28]. Thus, our detailed analysis of mitochondrial function and calculation of the coupling control efficiencies focused on the S pathway.

### Early Allograft Dysfunction, RTCA, HRR, and Histology

**FIGURE 2:**
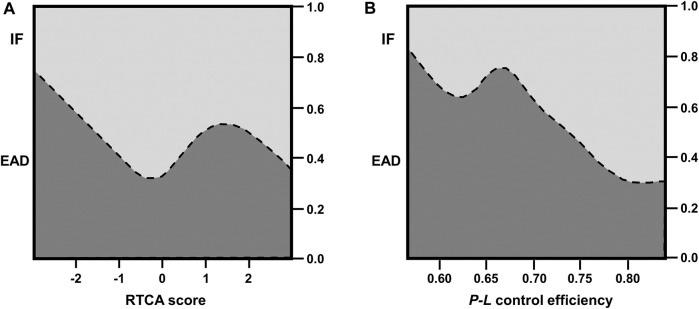
Conditional density plots of **(A)** RTCA score and **(B)** *P*-*L* coupling control efficiency of succinate pathway as factors impacting EAD.

**FIGURE 3:**
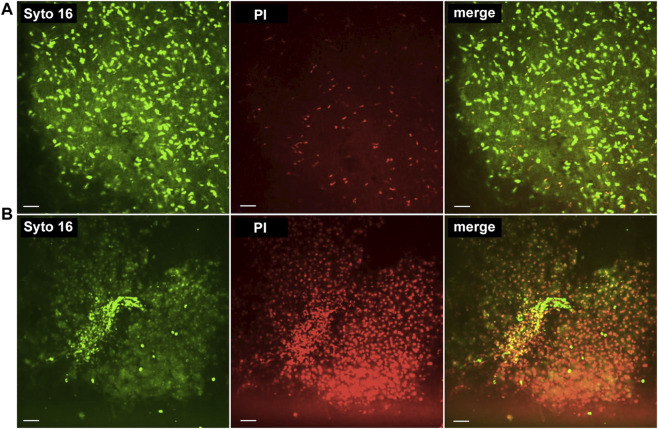
Real-time confocal microscopy. **(A)** RTCA score +3: 54-year-old female, non-steatotic liver; cause of death was traumatic head injury; cold ischemia time 8 h 12 min. Liver recipient did not experience early allograft dysfunction. **(B)** RTCA score −3: 53-year-old, female, non-steatotic liver; cause of death was intracerebral bleeding; cold ischemia time 9 h 32 min. Liver recipient suffered early allograft dysfunction. We used the following stains: i) Syto16^®^, which stains nuclei in dead and living cells, ii) propidium iodide (PI), which is only taken up by nuclei of dead cells; the merged image demonstrates the relation of stained cells within the tissue.

The assessment of RTCA and *P*-*L* coupling control efficiency revealed significant differences between EAD and IF livers: The mean RTCA score was significantly lower in the EAD cohort (0.75 ± 2.27 compared to 0.70 ± 2.08 in the IF cohort; *p* = 0.01), indicating a decreased cell viability. In agreement with the RTCA results, the *P*-*L* control efficiency was significantly better and predictive of IF (mean *P*-*L* control efficiency of 0.76 ± 0.06 in IF-livers vs. 0.70 ± 0.08 in EAD-livers; *p* = 0.02; Table 2; Figures 2, 3). The MEAF score correlated negatively with the RTCA score; *p* = 0.01, Spearman’s rho correlation coefficient −0.407; a lower viability correlated with a higher risk of liver dysfunction. Nonparametric correlation analysis showed that RTCA and *P*-*L* control efficiency are closely linked: *p* = 0.005, Spearman’s rho correlation coefficient was 0.493. When RTCA score and *P*-*L* control efficiency were adjusted for recipient and donor age as strongest confounders, the significance of both RTCA and OXPHOS coupling was confirmed (Table 3).

**TABLE 2:** Demographics and transplant factors of liver transplant recipients with analyzed biopsies (RTCA, HRR, histology).

	EAD (*N* = 22)	IF (*N* = 21)	*p*-value
**Characteristics**			
Donor age, [y] (mean ± SD)	52.45 ± 15.46	48.33 ± 15.65	0.40
**Donor BMI [kg·m** ^−**2**^ **] (mean ± SD)**	**28.05 ± 6.29**	**24.60 ± 4.16**	**0.03**
Extended criteria donor (ECD)—(*N*, %)	18 (81.8%)	13 (61.9%)	0.27
Age >65 years	5 (27.8%)	2 (15.4%)	
BMI >30 kg·m^−2^	4 (22.2%)	2 (15.4%)	
Macrovesicular steatosis >30%	5 (27.8%)	1 (7.7%)	
ICU-stay >7 days	2 (11.1%)	1 (7.7%)	
Infection serology	2 (11.1%)	1 (7.7%)	
Hypernatremia (Na-peak >165 mEq·L^−1^)	1 (5.6%)	1 (7.7%)	
Aspartate aminotransferase >90 U·L^−1^	5 (27.8%)	5 (38.5%)	
Alanine aminotransferase >105 U·L^−1^	3 (16.7%)	4 (30.8%)	
Total bilirubin >3 mg·dL^−1^	3 (16.7%)	4 (30.8%)	
LDRI (mean ± SD)	1.60 ± 0.34	1.51 ± 0.28	0.33
ET-DRI (mean ± SD)	1.75 ± 0.42	1.60 ± 0.28	0.19
Recipient age [y] (median, min-max)	60.23 ± 10.39	59.24 ± 9.29	0.48
Recipient BMI [kg·m^-2^] (mean, SD)	26.61 ± 5.10	25.72 ± 5.26	0.59
Prior transplantation (*N*, %)	1 (4.6%)	2 (9.5%)	
MELD score (mean ± SD)	16.41 ± 7.41	18.80 ± 8.00	0.38
Cold ischemia time [h] (mean ± SD)	8.41 ± 1.99	7.82 ± 2.26	0.5
Anhepatic time [min] (mean ± SD)	61.05 ± 19.54	52.29 ± 16.03	0.05
**Anastomosis time [min] (mean ± SD)**	**47.64 ± 9.86**	**40.57 ± 5.92**	**0.02**
Length of hospital stay [days] (median, IQR)	18.5 (15, 23.5)	19 (14.5, 28.5)	0.61
ICU stay after LT [days] (median, IQR)	4.5 (3, 7)	4 (2.5, 9.5)	0.81
**RTCA score**	**−0.75 ± 2.27**	**0.70 ± 2.08**	**0.01**
High-resolution respirometry			
OXPHOS capacity S_*P*_ [pmol·s^−1^·mg wet mass^-1^]	40.76 ± 16.26	38.83 ± 11.19	0.94
Cytochrome *c* control efficiency, 1-S_*P*_/S_*Pc*_	0.17 ± 0.12	0.11 ± 0.09	0.12
*** P-L* control efficiency, 1*-*S**_***L***_ **/S**_***P***_	**0.70 ± 0.08**	**0.76 ± 0.06**	**0.02**
Histology	2.52 ± 0.68	2.85 ± 0.49	0.06
Necrosis	0.41 ± 0.96	0.33 ± 0.73	0.87
Steatosis	0.64 ± 0.85	0.43 ± 0.60	0.61
Inflammation	0.65 ± 0.59	0.30 ± 0.47	0.08
Fibrosis	none	none	n.a
Vasculitis	0.20 ± 0.41	0.20 ± 0.41	1.000

Statistically significant differences are bold.

**TABLE 3:** RTCA score and *P-L* control efficiency as factors impacting EAD—adjusted for age.

Model A	Wald	Odds ratio	95% CI	*p-value*
Recipient age	0.126	0.987	0.921–1.059	*0.723*
Donor age	0.846	1.024	0.974–1.076	*0.358*
**RTCA score**	**3.886**	**0.736**	**0.542–0.998**	***0.049***
**Model B**	**Wald**	**Odds Ratio**	**95% CI**	***p-value***
Recipient age	0.268	0.979	0.905–1.060	*0.610*
Donor age	0.070	0.994	0.948–1.041	*0.791*
***P-L* control efficiency 1-S**_***L***_ **/S**_***P***_	**3.918**	**0.000**	**0.000–0.893**	***0.048***

RTCA, real-time confocal analysis.

EAD, early allograft dysfunction.

Statistically significant differences are bold.

**FIGURE 4:**
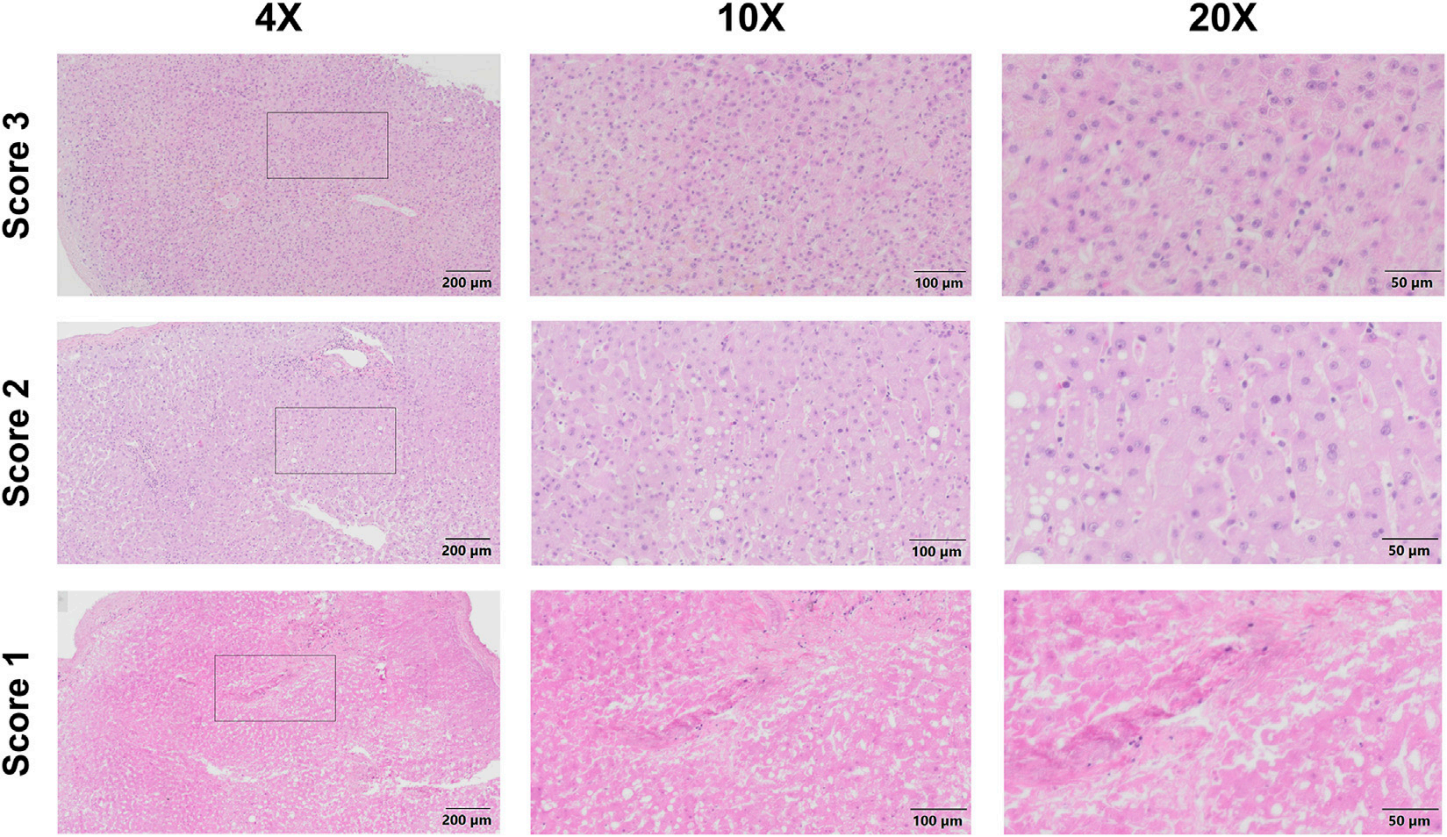
Histopathology of liver biopsy samples prior to liver transplantation. Representative images for overall histopathology score 3 (normal liver tissue, first row), score 2 (moderate changes including steatosis and mild periportal and parenchymal inflammation, second row), and score 1 (severe changes including extensive necrosis, third row) are shown.

In contrast, histology did not differ between EAD and IF, although there was a trend towards better overall scores in the IF group (Table 2; Figure 4). Accordingly, none of the individual histopathological features such as necrosis, steatosis, inflammation, and vasculitis correlated with the outcome.

### Graft and Patient Survival

The 90-day mortality was 7.0% (3/43; 2 after EAD); 90-day graft loss was 4.7% (2/43; 1 after EAD). Eight patients (8/43, 18.6%, 6 after EAD) died during the follow up; three patients (3/43, 7.0%, 2 after EAD) had to undergo a re-transplant within the first year after transplant. The succinate-linked OXPHOS capacity was predictive for patient survival in the univariate Cox regression analysis.

Overall graft loss and patient death were numerically higher in the EAD group, but not significantly different in comparison to IF livers; graft loss after EAD 4/22 (18.2%) vs. IF 1/21 (4.8%), *p* = 0.18; death after EAD 6/22 (27.3%) vs. 3/21 (14.3%), *p* = 0.31. Re-transplantation was the only risk factor independently predictive for graft survival in the univariate Cox regression analysis; *p* = 0.04, HR 19.3, Wald 4.4. Univariate and multivariate analyses for patient survival are displayed in Tables 4, 5. The most important and independent factor for patient survival was also re-transplantation; *p* = 0.001, HR 105.2, Wald 10.279.

**TABLE 4:** Univariate Cox regression analysis—patient survival.

Characteristic	Wald	HR	95% CI	*p-value*
**Recipient age [y]**	**4.43**	**0.943**	**0.892–0.996**	**0.035**
Recipient BMI [kg·m^−2^]	0.139	1.026	0.896–1.176	0.71
Recipient sex	0.547	0.553	0.115–2.662	0.46
BAR score	0.086	1.018	0.905–1.144	0.8
**Prior Tx**	**12.776**	**66.01**	**6.635–656.759**	**< 0.001**
Donor age in [y]	0.005	1.001	0.960–1.044	0.945
Donor BMI [kg·m^−2^]	0.02	0.991	0.882–1.115	0.887
Donor sex	0.114	0.797	0.214–2.973	0.736
Extended criteria donor (ECD)	1.282	3.326	0.415–26.637	0.258
ET-DRI	3.269	3.56	0.899–14.100	0.071
LDRI	2.347	5.469	0.622–48.090	0.126
Cold ischemia time [h]	1.734	1.004	0.998–1.009	0.19
Anastomosis time [min]	0.451	1.023	0.958–1.093	0.5
RTCA score	1.236	1.196	0.872–1.641	0.266
**Succinate-linked OXPHOS capacity S**_***P***_ **[pmol**·**s** ^−**1**^·**mg wet mass** ^−**1**^ **]**	**4.955**	**1.054**	**1.006–1.104**	**0.03**
*P*-*L* control efficiency 1-S_*L*_/S_*P*_	1.344	0.003	0.000–56.131	0.25
Steatosis in zero biopsy	0.268	1.237	0.554–2.764	0.6
Necrosis in zero biopsy	0.332	0.742	0.267–2.054	0.6
EAD	0.768	1.859	0.464–7.440	0.381
**Graft loss**	**8.995**	**7.657**	**2.025–28.958**	**0.003**

HR, hazard ratio; CI, confidence interval; BAR, balance of risk; ET DRI, eurotransplant donor risk index; LDRI, liver donor risk index; RTCA, real-time confocal analysis.

Statistically significant differences are bold.

**TABLE 5:** Multivariate Cox regression analysis—patient survival.

Characteristic	Wald	HR	95% CI	*p-value*
Recipient age [y]	0.624	0.965	0.884–1.054	0.429
**Prior transplantation**	**10.279**	**105.188**	**6.108–1811.585**	**0.001**
Succinate–linked OXPHOS capacity S_*P*_ [pmol·s^−1^·mg wet mass^-1^]	0.209	1.018	0.944–1.097	0.65
Graft loss	2.724	6.494	0.704–59.878	0.099

HR, hazard ratio; CI, confidence interval.

Statistically significant differences are bold.

## Discussion

In this prospective clinical pilot trial, we assessed liver biopsies using HRR and RTCA during SCS. We found both methods applicable, clinically feasible and more meaningful for the short-term outcome after LT when compared to standard haematoxylin and eosin histology of pre-implantation biopsies. Our approach with analysis during SCS was designed to mimic a pre-transplant decision-making process, similar as aided by routine frozen section histology.

Martins and co-workers previously evaluated mitochondrial function as a possible tool to determine graft quality before LT [15]. Their comprehensive study design included measurement of mitochondrial respiration, mitochondrial membrane potential, and intracellular ATP content. In the present clinical trial, we aimed at validating HRR as a rapid assay requiring only a small tissue sample mass. We found that *P*-*L* control efficiency of the succinate pathway measured before transplantation correlates with EAD. The results of our study are based on a limited number of liver transplants which calls for caution in the interpretation of statistical tests. However, mitochondrial function was aligned with the RTCA score and correlated with the clinical endpoints, indicating that we are measuring a true and relevant phenomenon.

Recently, Weissenbacher *et al.* [18] published the predictive value of the RTCA score for delayed graft function (DGF) in kidney pre-implantation biopsies. The added value and information of the RTCA in this study is the quantification of the acute and ischemia-related cellular damage in addition to pre-existing injury as characterized by histology. Similar to kidney transplantation, standard histology failed to predict the initial function in LT. The degree of steatosis and necrosis in the pre-implantation biopsy did not correlate with early allograft function. While this might be attributable to the limited sample size, it also relates to the fact that microscopic structural damage is a parameter with limited value towards the decision to transplant or discard an individual organ. The added value of RTCA together with mitochondrial assessment is due to the fact, that these techniques measure, display and quantify the additional acute injury at cellular and subcellular levels.

For HRR, only 2 mg of sample (wet mass) per measurement were required. This is an order of magnitude less when compared to a recent study employing other methods for functional assessment of mitochondrial respiration [15] and speaks towards the feasibility of this approach in clinical settings. Instead of time-consuming isolation of mitochondria for HRR, a liver homogenate was prepared using a tissue shredder. This preparation method takes less than 5 min, requires a small sample size, and contains all mitochondrial subpopulations [30]. Mitochondrial respiration was assessed at 37°C, ruling out temperature-dependent deviations for the extent and mechanism of mitochondrial coupling.

The *P*-*L* control efficiency (ratio of net to total OXPHOS capacity) for the succinate pathway was calculated and used as a statistically more robust parameter compared to the classical respiratory acceptor control ratio (RCR), the State 3/State 4 flux ratio [28]. Importantly, the OXPHOS state is defined by saturating ADP and inorganic phosphate concentrations while State 3 only indicates high ADP and inorganic phosphate concentrations, which are not necessarily saturating.

While RTCA and mitochondrial function were predictive of postoperative organ function, they were not predictive for graft or patient survival. In contrast, the OXPHOS capacity of the mitochondrial succinate pathway was found predictive of patient survival in the univariate analysis. This parameter is closely related to the tissue viability and mitochondrial mass concentration. Recently, our group demonstrated the predictive value of mitochondrial respiratory capacity in a setting of clinical NMP and LT [20]. In agreement with the published trial, we herein establish the feasibility and value of a functional mitochondrial measurement in standard cold storage and LT.

Whereas the assessment requires fresh tissue, the considerable advantage of HRR and RTCA is the rapid process. For HRR, the tiny tissue sample is rewarmed to 37°C and resupplied with oxygen, mimicking physiological temperatures, thus enabling mitochondrial performance testing despite sampling during SCS.

The high percentage of EAD in the cohort is primarily not a result of a bias due to the relatively low number of transplanted livers. As demonstrated by Fodor *et al.* [31], the rate of EAD in our center raised over the last years, mainly because of increasing acceptance of ECD grafts. Indeed, in the present study cohort, 72% of the liver grafts stemmed from ECD donors. Recent developments with pre-transplant machine perfusion are promising ways to reduce EAD.

In summary, tissue analysis by RTCA and HRR shed light into viability and bioenergetic performance of SCS liver allografts and can be applied to anticipate EAD. Our results further enhance the understanding and relevance of bioenergetic function in liver ischemia and transplantation and provide the basis for further consideration of these parameters as biomarkers in LT. These observations confirm previous studies and serve to underline the feasibility of RTCA and mitochondrial functional tests as tools for liver quality assessment prior to transplantation [15].

## Data Availability

The original contributions presented in the study are included in the article/Supplementary Material, further inquiries can be directed to the corresponding authors.

## Footnotes

1
https://wiki.oroboros.at/index.php/SUIT-025

2
https://wiki.oroboros.at/index.php/SUIT-006

## Glossary

ALT: alanine aminotransferase
AST: aspartate aminotransferase
ATP: adenosine triphosphate
BAR: balance of risk
BMI: body mass index
DAMP: damage associated molecular pattern
DCD: donation after circulatory death
EAD: early allograft dysfunction
ECD: extended criteria donors
ET-DRI: Eurotransplant donor risk index
F: FAO pathway
FAO: fatty acid oxidation
*FCR*: flux control ratio
FMN: flavin mononucleotide
HOPE: hypothermic oxygenated machine perfusion
HR: hazard ratio
HRR: high-resolution respirometry
IF: initial function
IRI: ischemia-reperfusion injury
*L*: LEAK respiration
LDRI: Liver donor risk index
LEAK: the state of non-phosphorylating resting respiration in the absence of ADP
L-GrAFT: Liver Graft Assessment Following Transplantation
LT: liver transplantation
MEAF: Model for Early Allograft Function
N: NADH-linked pathway
OXPHOS: oxidative phosphorylation
*P*: respiratory OXPHOS rate in the presence of kinetically saturating ADP
PI: propidium iodide
ROS: reactive oxygen species
RTCA: real-time confocal imaging analysis
S: succinate-linked pathway
SCS: static cold storage
SUIT: Substrate-Uncoupler-Inhibitor Titration
WGA: wheat germ agglutinin
