# Supplementary material for: The Predictive Value of Graft Viability and Bioenergetics Testing Towards the Outcome in Liver Transplantation
Source: Transpl Int. 2024 Feb 23;37:12380. doi: 10.3389/ti.2024.12380 (PMC10920229; doi:10.3389/ti.2024.12380)
Supplement: Supplementary file 1 [file DataSheet1.docx]

**Supplement**

**Supplementary table 1: SUIT protocol I**

| Step | Compound | Concentration | Coupling control state | ET-pathway state | Function in the protocol |
| --- | --- | --- | --- | --- | --- |
| 1 | Tissue homogenate (2 mL final volume) | 1 mg wet mass·mL^-1^ | REN | Residual endogenous respiration |  |
| 2 | ADP · Mg^2+^ (Merck, 117105) | 5 mM | REN |  | Substrate of ATP synthase |
| 3 | Malate (Sigma, M1000) | 0.1 mM |  |  | Required to generate oxaloacetate which reacts then with acetyl-CoA |
| 4 | Octanoylcarnitine (APExBIO, B6371) | 0.5 mM | OXPHOS (*P*) | FAO | Substrate for fatty acid oxidation (FAO) |
| 5 | Cytochrome *c* (Sigma, C7752) | 10 µM | OXPHOS (*P*) | FAO | Evaluate mitochondrial outer membrane integrity |
| 6 | Pyruvate (Sigma, P2256) | 5 mM | OXPHOS (*P*) | FAO+NADH | Feeds TCA (via Acetyl-CoA) and supports NADH-linked pathway |
| 7 | Malate (Sigma, M1000) | 2 mM | OXPHOS (*P*) | FAO+NADH | Supports NADH-linked pathway |
| 8 | Glutamate (Sigma, G1626) | 10 mM | OXPHOS (*P*) | FAO+NADH | Supports NADH-linked pathway |
| 9 | Succinate (Sigma, S2378) | 10 mM | OXPHOS (*P*) | FAO+NADH+S | Substrate of Complex II |
| 10 | Rotenone (Sigma, R8875) | 0.5 µM | OXPHOS (*P*) | S | Inhibition of Complex I |
| 11 | Antimycin A (Sigma, A8674) | 2.5 µM | ROX | Residual oxygen consumption | Inhibition of Complex III |

**Supplementary table 2: SUIT protocol II**

| Step | Chemical | Concentration | Coupling control state | Function in the protocol |
| --- | --- | --- | --- | --- |
| 1 | Tissue homogenate (2 mL final volume) | 1 mg wet mass·mL^-1^ | REN |  |
| 2 | Rotenone (Sigma, R8875) | 0.5 µM | ROX | Inhibition of Complex I |
| 3 | Succinate (Sigma, S2378) | 10 mM | LEAK (*L*) | Substrate of Complex II |
| 4 | ADP · Mg^2+^ (Merck, 117105) | 5 mM | OXPHOS (*P*) | Substrate of ATP synthase |
| 5 | Cytochrome *c* (Sigma, C7752) | 10 µM | OXPHOS (*P*_c_) | Evaluate mitochondrial outer membrane integrity |
| 6 | Antimycin A (Sigma, A8674) | 2.5 µM | ROX | Inhibition of Complex III |

**Supplementary table 3: Histopathologic scoring of liver biopsies**

|  | **Necrosis** | **Steatosis** | **Inflammation** | **Fibrosis** | **Vessels/sinusoids** |
| --- | --- | --- | --- | --- | --- |
| **Score 0** | none | none | none | none | normal |
| **Score 1** | < 20 % | < 40 % | mild, spotted portal and/or lobular | < 20 % | dilated sinusoids |
| **Score 2** | > 20-50 % | > 40-80 % | moderate portal and/or lobular | > 20-50 % | vasculopathy with luminal constriction |
| **Score 3** | > 50 % | > 80 % | dense portal and/or lobular | > 50 % | vasculitis |
